# Analysis of brain functional connectivity in children with autism spectrum disorder and sleep disorders: a fNIRS observational study

**DOI:** 10.3389/fpsyg.2025.1544798

**Published:** 2025-05-09

**Authors:** Fengli Bi, Zhuting Jia, Lin Lv, Yanyan Zhang, Chuanhua Zhu, Chunxiao Wan

**Affiliations:** ^1^Department of Physical and Rehabilitation Medicine, Tianjin Medical University General Hospital, Tianjin, China; ^2^Department of Emergency, Binzhou Medical College Affiliated Hospital, Binzhou, China; ^3^Department of Rehabilitation, Baotou Central Hospital, Baotou, China; ^4^Department of Paediatrics, Binzhou Medical College Affiliated Hospital, Binzhou, China; ^5^Department of Rehabilitation, Binzhou Medical College Affiliated Hospital, Binzhou, China

**Keywords:** autism spectrum disorder, fNIRS, sleep disorder, resting-state, functional connectivity

## Abstract

**Introduction:**

Autism spectrum disorder (ASD) is often associated with sleep disorders, although the neurophysiological reasons behind these issues are poorly understood. In this cross-sectional study, functional near-infrared spectroscopy (fNIRS) was used to compare differences in brain functional connectivity (FC) in children with ASD and sleep disorders and those with ASD that was not complicated by sleep disorders.

**Methods:**

A total of 88 children (4–9 years old, either sex) were included in the study. The children were divided into three groups: those with ASD and sleep disorders (ASD with sleep disorder group; *n* = 29), those with ASD and no sleep disorders (ASD without sleep disorder group; *n* = 29), and those with typical development (TD group; *n* = 30). All children with ASD met the diagnostic criteria for the “Diagnostic and Statistical Manual of Mental Disorders-5 (DSM-V).” The ASD group with sleep disorders showed typical sleep disorder symptoms, with a total score of ≥41 on the Children’s Sleep Habits Questionnaire. All children were assessed using the Autism Diagnostic Observation Scale, the Vineland Adaptive Behavior Scale, third edition, the Social Response Scale, and the Children’s Sleep Habits Questionnaire. The fNIRS detection was conducted in a quiet environment.

**Results:**

The fNIRS data revealed that under resting-state conditions, the supramarginal gyrus [SMG:Cohen’s *f* = 0.981(L)*f* = 0.467(R)], inferior frontal gyrus [IFG:Cohen’s *f* = 0.415(L)*f* = 0.443(R)], frontopolar area [FPA:Cohen’s *f* = 0.620(L)*f* = 0.634(R)], dorsolateral prefrontal cortex [DLPFC:Cohen’s *f* = 0.593(L)*f* = 0.547(R)], and visual association cortex [VAC:Cohen’s *f* = 0.500(L)*f* = 0.524(R)] of the brain showed lower activity in ASD with sleep disorder group compared with the TD group (*p* < 0.01). The FC values for the SMG [Cohen’s *f* = 0.981(L)*f* = 0.467(R)], RFPA (Cohen’s *f* = 0.634), DLPFC [Cohen’s *f* = 0.593(L)*f* = 0.547(R)], and VAC [Cohen’s *f* = 0.500(L)*f* = 0.524(R)] were also lower in the ASD with sleep disorder group than the ASD without sleep disorder group (*p* < 0.01). The FC values of the LIFG showed a mild negative correlation with social affect scale scores (r = −0.34, *p* = 0.07), while FC values in the RDLPFC were negatively correlated with restricted repetitive behavior (RRB) (r = −0.41, *p* = 0.03). The Children’s Sleep Habits Questionnaire scores showed a positive correlation with FC values in the RIFG region of the brain (r = 0.37, *p* = 0.05).

**Conclusion:**

The results indicate that FC in the resting brain of children with ASD complicated with sleep disorders was weaker than that of children with ASD without sleep disorders. Both groups showed weaker FC compared with the TD group. However, due to the limited sample size, the generalizability of the findings requires further validation in multicenter, large-sample studies.

## 1 Introduction

Autism spectrum disorder (ASD) is a developmental disorder characterized by limited interest, repetitive behavior patterns, and persistent deficits in social interaction and communication skills (Keepers et al., 2020). According to recent statistics, the global prevalence of autism is approximately 1%, with the percentage increasing every year (Zeidan et al., 2022). According to CDC statistics released in March 2023, 1 in every 36 children aged 8 years has ASD, with boys accounting for 4% and girls for 1%. As a result, ASD has emerged as the leading cause of disability in children with mental disorders worldwide. Moreover, approximately 40%–80% of children with ASD have sleep disorders, which mainly include delayed sleep onset, night-time awakenings, parasomnia, sleep-disordered breathing, and daytime sleepiness (Yao et al., 2022). In comparison to children with ASD and no sleep disorder, ASD children with sleep disorders exhibit critical language disorders, social disorders, problematic behaviors, and emotional issues, making rehabilitation difficult.

Functional near-infrared spectroscopy (fNIRS) is a non-invasive neural activity imaging technique that operates on the optical principle and utilizes the near-infrared spectrum of light (650–950 nm) to penetrate biological tissues (Wyatt et al., 1986). The present study examined FC, oxygenated hemoglobin (HbO2), deoxygenated hemoglobin (HbR), total hemoglobin concentration, and the distribution and changes in blood volume and oxygen saturation in the cerebral cortex, to determine the level of brain activity. These measures effectively reflect neuronal activity, the degree of activation in different brain regions, and the strength of neural connections. The advantages of fNIRS include its non-invasiveness, portability, low cost, relative insensitivity to head movements, and relatively high spatial resolution capture, all of which have led to its widespread application for the measurement of the activation state of brain function in autism (Yanagisawa et al., 2016), hyperactivity disorder (Yasumura et al., 2014), and infants (Behrendt et al., 2020; Karen et al., 2019).

The study investigated the association between FC and ASD-associated characteristics such as social communication deficits and sleep disorders. The objective of the present study was to use fNIRS to better understand the FC of the resting brain in children with ASD accompanied by sleep disorders compared with ASD without sleep disorders and TD children. Several mechanistic studies on children with ASD have suggested that they exhibit resting-state functional connectivity (RSFC) anomalies. A study (Li and Yu, 2016) reported that the RSFC between the bilateral temporal lobes in children with ASD was weaker than in healthy children, and the former also had a larger fluctuation range of HbO_2_ and HbR. Sperdin and Schaer’s near-infrared studies revealed insufficient connections between the middle superior temporal sulcus and bilateral ventral tegmental regions in children with ASD (Sperdin and Schaer, 2016). Other regions, such as the pontine nucleus, the left hemispherical prefrontal lobe, and the orbitofrontal and ventromedial prefrontal cortex, show insufficient connectivity. Yanwei Li used fNIRS to study the functional network efficiency of children with ASD and reported a weak connection between the bilateral prefrontal cortex and the bilateral temporal cortex (Li and Yu, 2016).

Functional near-infrared spectroscopy (fNIRS) has been used to detect hemodynamic changes in different regions of the cerebral cortex in children with ASD, with a focus on developmental abnormalities in the brain during rest and task states, and their associations with various neuropsychological functions and clinical manifestations. Ayelet Arazi et al. found that disruption of sleep homeostasis regulation *in vivo* may lead to sleep disturbances in children with ASD and is indicative of weaker sleep pressure, as evidenced by electroencephalography (EEG) studies. The lateral hypothalamus, a brain region involved in promoting and maintaining arousal, has also been implicated in ASD. Functional magnetic resonance imaging (fMRI) studies in children with ASD revealed reduced functional connectivity (FC) between the amygdala and other brain regions, which may lead to prolonged arousal in these patients. Another fMRI study found that thalamo-cortical FC was dysregulated in children with ASD, leading to an abnormal increase in thalamo-cortical FC and resulting in sleep disorders. Adams et al. discovered the correlation between the severity of sleep disturbances in ASD patients and the severity of core symptoms. This study hypothesizes that children with ASD and sleep disorders have more severe social and behavioral issues, and that greater developmental abnormalities in the cerebral cortex lead to weaker FC between various brain regions. Functional near-infrared spectroscopy (fNIRS) is more commonly used in the research of children with ASD, particularly in quiet and social interaction settings, due to its portability and lower sensitivity to movement. Compared to fMRI, fNIRS gives better temporal resolution, but EEG provides superior spatial resolution. Thus, fNIRS was selected for monitoring brain function in this study. Based on previous research, this study systematically explores the FC characteristics of ASD children with sleep disorders in the resting state of the brain, revealing potential associations with sleep disturbances and core symptoms and providing clinical evidence for a deeper understanding of neurodevelopmental heterogeneity in children with ASD.

## 2 Methods

### 2.1 Participants

**Figure 1:**
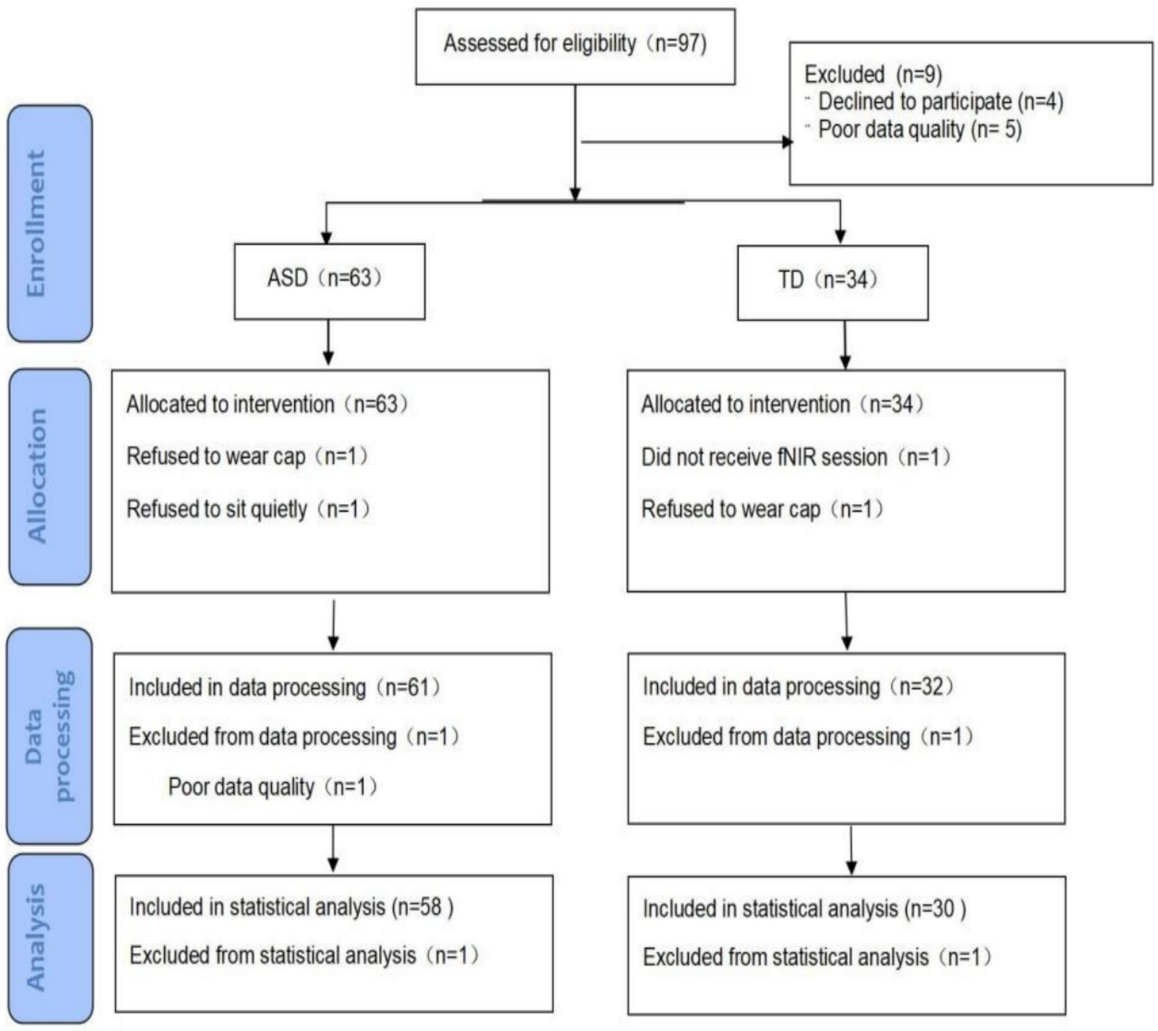
Details of attrition from enrollment to data analysis in the ASD and TD groups.

The registration period began in April 2024, with follow-ups lasting 3 months after the end of the assessment. The study initially enrolled 97 children, nine of whom were excluded from the analysis due to inconsistencies at baseline and demographic differences; the reasons for exclusion are shown in Figure 1. The study eventually enrolled 58 children with ASD (including 29 with and 29 without sleep disorders) and 30 children with TD. All of the children with ASD met the DSM-V diagnostic criteria for the American Psychiatric Association (2013), which was confirmed using the Autism Diagnostic Observation Schedule (Lord et al., 2000). All children with ASD were recruited from the Children’s Rehabilitation Center of the Binzhou Medical College Affiliated Hospital. Children with typical development (TD) were recruited from nursery and primary schools, the Children’s Rehabilitation Center, and through social media. All assessments were conducted by a certified ADOS-2 (Autism Diagnostic Observation Schedule, Second Edition) assessor with an official ADOS-2 research-level credential. To ensure assessment consistency, standardized operational procedures (SOPs) were followed throughout the process. ASD diagnoses were validated using the standardized ADOS thresholds. To minimize sampling bias, age/sex-based stratified randomization was used. The protocol was approved by the Research Ethics Committee of the Affiliated Hospital of Binzhou Medical College (protocol number: KYLL-060).

The inclusion criteria for the ASD with sleep disorder group were as follows: (1) aged 4–9 years old, of either sex; (2) right-handed; (3) the presence of typical clinical symptoms, such as sleep resistance, difficulty falling asleep, late sleeping, frequent night waking, night terrors, excessive early waking, short sleep times, circadian rhythm disturbances, and daytime sleepiness, among others; (4) none of the children had taken exogenous melatonin, antipsychotics, or hypnotics within the past month; and (5) a total score of Children’s Sleep Habits Questionnaire ≥ 41.

The exclusion criteria for the ASD group were as follows: (1) other neurological diseases; (2) ASD-associated chromosomal or genetic abnormalities; (3) epilepsy and use of anti-seizure medications; (4) Organic diseases of the respiratory system, including abnormal anatomical structure (such as airway stenosis and tonsil hypertrophy), and sleep-related breathing disorders caused by local tissue compliance problems; and (5) inability to complete the test.

The inclusion criteria for the TD group were as follows: (1) Absence of any neurological or developmental diagnosis/delay, preterm birth, or other significant birth history; (2) no neurological or psychotropic drug use; (3) no seizures; (4) no family history of ASD; and (5) no clinical manifestations of sleep disorders.

### 2.2 Experimental protocol

In this experiment, NirScan-6000A equipment (Danyang Huichuang Medical Equipment Co., Ltd., China) was used to continuously measure and record the concentration changes of brain oxygenated hemoglobin (HbO) and deoxy hemoglobin (HbR) during the task. The system consists of a near-infrared light source (light emitting diodes, LEDs) and avalanche photodiodes (APDs) as detectors, with wavelengths of 730, 808, and 850 nm, respectively, with a sampling rate of 11 Hz. The experiment uses 24 light sources and 16 detectors to form 63 effective channels; the average distance between the source and the detector is 3 cm (range 2.7–3.3 cm). Previous studies have demonstrated abnormal frontal cortex development in individuals with ASD (Carper and Courchesne, 2005). In such cases, only the frontal lobe shows significant between-group differences, with diminished information transfer within the frontal lobe, temporal lobe, cerebellum, and limbic system (Fu et al., 2025). Shen et al. (2016) observed decreased connectivity between the primary visual cortex and sensorimotor regions.

So, based on the anatomical location of Brodmann’s region and the participant’s cortical region (Zhang et al., 2021), the ROI was divided into the right and left supramarginal gyrus, the inferior frontal gyrus, the frontal polar region, the dorsolateral prefrontal cortex, and the visual association cortex, and ROI-to-ROI connectivity was performed. The NIR function was measured when the participant was seated separately in a quiet room. Each participant was asked to sit quietly for 8 min, with no head movements, eyes closed, no sleeping, and keeping the surrounding environment quiet.

### 2.3 fNIRS data processing

The Preprocess module of NirSpark software (Huichuang, China) was used for the preprocessing of all collected data. ① The first 30 s of data were removed to stabilize the signal, after which the spline interpolation algorithm was applied to identify and remove the motion artifacts, and the signal standard deviation threshold was set to 6, and the peak threshold was set to 0.5. Specifically, the standard deviation (STD) of each segment of the signal is calculated using the sliding window method, and smaller STD values are averaged to establish thresholds for subsequent artifact identification. Once the artifacts are identified, cubic spline interpolation (Spline) is applied to correct for baseline drift, and spike noise is replaced with Gaussian white noise. This method effectively removes various motion artifacts, including baseline drift, spikes, and continuous interference, thereby enhancing the quality of fNIRS data (Iester et al., 2024; Yang et al., 2022). ② The remaining data were subjected to band-pass filtering (0.009–0.08 Hz) (Cui et al., 2011; Sasai et al., 2011; Zhu et al., 2014) to eliminate high-frequency noise from heartbeat, respiration, and Mel wave, as well as low-frequency drift, instrument noise, and blood pressure fluctuations; ③ According to the revised Bier-Lambert law, the relative concentration of oxygenated hemoglobin in each channel was calculated. Our approach, which is based on the ROI-ROI methodology, aims to improve the signal-to-noise ratio. These included ROI1 covering the superior limbic gyrus, corresponding to channels 1, 13, 15, 48, and 52, ROI2 representing the inferior frontal gyrus and corresponding to channels 3, 4, 11, and 12, ROI3 covering the frontal pole area and corresponding to channels 5, 6, 7, 8, 9, 10, 18, 20, 22, 23, and 25, ROI4 representing the dorsolateral prefrontal cortex, corresponding to channels 19, 24, 26, 29, and 34, and ROI5 consisting of the visual association cortex, corresponding to channels 42, 43, 58, 59, 60, 61, 62, and 63. The Pearson’s correlation coefficient r value of oxyhemoglobin on the ROI-ROI time series was calculated, and the correlation coefficient r value was transformed by Fisher-z transformation so that the correlation coefficient conformed to the normal distribution. The transformed z value was used as the connection strength of each ROI-ROI to compare the relative connection strengths of each ROI.

### 2.4 Behavioral measurements

The parents of each participant were asked to sign an informed consent form for the fNIRS examination. All scale assessments were performed by the same investigator. The parents of the ASD and TD children were assessed for behavioral development using the Vineland Adaptive Behavior Scale (VABS-3), the Social Responsiveness Scale (SRS-2), and the Children’s Sleep Habits Questionnaire (CSHQ).

The autism diagnostic observation scale-the second edition (The Autism Diagnostic Observation Schedule, ADOS-2) is a standardized and semi-structured assessment tool to determine the diagnostic criteria and severity of autism. The evaluator may select the appropriate assessment module that is suitable for the development and language level of the subject according to their ability. The ADOS and Autism Diagnostic Interview-Revised (ADI-R) are considered the gold standard tools for the diagnosis of ASD. It facilitates the professional qualification certification of subjects for their ADOS-2 diagnostic evaluation, as well as to obtain social influence (Social Affect, SA) scores and repeat stereotyped behavior (Restricted Repetitive Behavior, RRB) scores. These scores are then converted to the standardized severity score (CSS). The standard scores indicate the severity of autism.

The Vineland Adaptive Behavior Scales Third Edition (VABS-3) is an adaptive behavior measurement tool used to assess an individual’s personal and social adequacy (Ludwig et al., 2023) during daily activities. This scale evaluates the five aspects, such as communication ability, daily life skills, socialization range, motor skills range, and bad behavior range (Nickerson et al., 2019).

The social response scale-the second edition (SRS-2) is used mainly for evaluating the social ability of individuals with autism to reveal the association between the abnormal social behavior of the subject and the difficulty encountered during everyday social behavior. The aim is to provide a basis for the clinical diagnosis or a psychological education plan. The Chinese Mandarin version of the SRS-2 scale was validated and found to be reliable. The SRS-2 consists of five aspects: social awareness, social cognition, social communication, social motivation, and behavioral pattern, with scores ranging from “never” to “always,” corresponding to scores of 0 to 3, with a total score of 0–195. Higher scores indicate a more severe impairment of social communication.

The Children’s Sleep Habits Questionnaire (CSHQ) facilitates the evaluation of sleep disorders in children aged 4 to 10 from the following perspectives: resistance, sleep latency, late sleep, anxiety, night awakenings, parasomnias, sleep apnea, and daytime sleepiness. A total score of ≥41 in CSHQ indicates the existence of sleep disorders, with a higher score representing a more evident sleep disorder. CSHQ is widely used in the assessment of sleep disorders in children with ASD.

### 2.5 Statistical analysis

The sample groups are approximately independent, without significant bias, and the sample size is small. After conducting the Kolmogorov–Smirnov test, skewness and kurtosis were found to be within an acceptable range, indicating that the data do not significantly deviate from normality. Therefore, the data are described using the mean ± standard deviation. When comparing two groups of quantitative data, a two-independent sample t-test was used if the variance was homogeneous; if the variance was not homogeneous, a corrected t-test was applied. For comparing quantitative data across multiple groups, one-way ANOVA was used when the samples in each group were normally distributed and the variance was homogeneous. If the samples were normally distributed but the variance was not homogeneous, the Kruskal–Wallis test was used for comparison. Pearson’s correlation coefficient (r) was used to assess the linear relationship between FC for brain regions and clinical scale scores, while Spearman’s rank correlation (*ρ*) was used for non-normally distributed data. A *p*-value of <0.05 was considered statistically significant. After comparison, the Bonferroni method was used to adjust the inspection level. All analyses were performed using Stata17.0.

## 3 Results

### 3.1 Demographics

Figure 1 provides details of missing participants, including the reasons for exclusion and dropout rates. Data on 58 children with ASD and 30 children with TD were available for analysis.

The study included 88 participants: 30 children in the normal (TD) group, 29 in the ASD without sleep disorder group, and 29 in the ASD with sleep disorder group. Table 1 shows the sex and age distributions of the children in the three groups; there were no significant differences among the groups (*p* > 0.05). The skewness and kurtosis of the variables were found to be within reasonable limits, indicating that the data were essentially normally distributed. Skewness reflects the symmetry, while kurtosis describes sharpness in the data distribution. Skewness values between −2 and 2 and kurtosis values between −7 and 7 are considered close to the criteria for a normal distribution. Therefore, the current data met the normality assumption and may provide a valid basis for subsequent statistical analysis.

**Table 1:** Basic information on the research subjects.

Brain area	TD group (30)	Group of ASD without sleep disorder (29)	Group of ASD with sleep disorder (29)	Statistics	*p*-value	Cohen’s d/*f*
Gender						
Female	8 (26.67%)	3 (10.34%)	7 (24.14%)	2.78	0.250	
Male	22 (73.33%)	26 (89.66%)	22 (75.86%)			
Age	6.20 ± 1.243	5.72 ± 1.131	5.97 ± 1.614	0.92	0.401	0.147
Social Affect (SA)	2.00 ± 1.20	16.76 ± 4.70	17.93 ± 3.87^*^	184.14	<0.001	2.081
Restricted repetitive behavior (RRB)	0.63 ± 0.72	4.93 ± 2.40	4.72 ± 2.93^*^	35.55	<0.001	0.915
Raw scores	2.63 ± 1.50	21.69 ± 6.31	22.66 ± 5.68^*^	154.24	<0.001	1.905
Standardized scores	1.63 ± 0.56	8.69 ± 1.65	8.86 ± 1.57^*^	277.38	<0.001	2.555
Communication (COM)	39.07 ± 6.86	20.55 ± 11.13^*^	21.03 ± 9.86^*^	37.15	<0.001	0.935
Daily living skills (DLSs)	37.07 ± 6.18	25.55 ± 6.27^*^	26.93 ± 5.49^*^	32.66	<0.001	0.877
Socialization (SOC)	43.37 ± 6.08	13.00 ± 5.95^*^	16.41 ± 7.08^*^	200.99	<0.001	2.175
Social reactivity Scale (SRS)	47.63 ± 4.96	69.90 ± 14.40^*^	70.24 ± 9.54^*^	46.68	<0.001	1.048
Children’s sleep habits questionnaire (CSHQ)	30.27 ± 5.56	29.79 ± 5.57	57.17 ± 3.23^*#^	295.97	<0.001	2.639

^*^Indicates a comparison with the TD group, *p* < 0.01; ^#^indicates a comparison with the ASD without sleep disorder group, *p* < 0.01.

The statistical analysis found no statistical differences in social emotion (SA), restrictive repetitive behavior (RRB), total score, and standard score between the two groups of children with ASD (*p* > 0.05). However, significant differences were noted in the scores of communication (COM), daily living skills (DLS), social ability (SOC), social response scale, and Children’s Sleep Habits Questionnaire (*p* < 0.001).

Further comparison found that the communication (COM), daily living skills (DLS), and social ability (SOC) scores of children were higher in the TD group than in the ASD group, with no difference noted between the two ASD groups with and without sleep disorders. The social response scale scores were lower in the TD group than in the two ASD groups, whereas there was no difference between the two ASD groups. The scores on the Children’s Sleep Habits Questionnaire did not differ between the TD and ASD without sleep disorder group, and both were lower than the ASD with sleep disorder group. There were significant differences in effect sizes for each indicator, except for age/sex (Cohen’s *f* > 0.4).

### 3.2 Comparison of FC under resting-state conditions among the three groups

**Figure 2:**
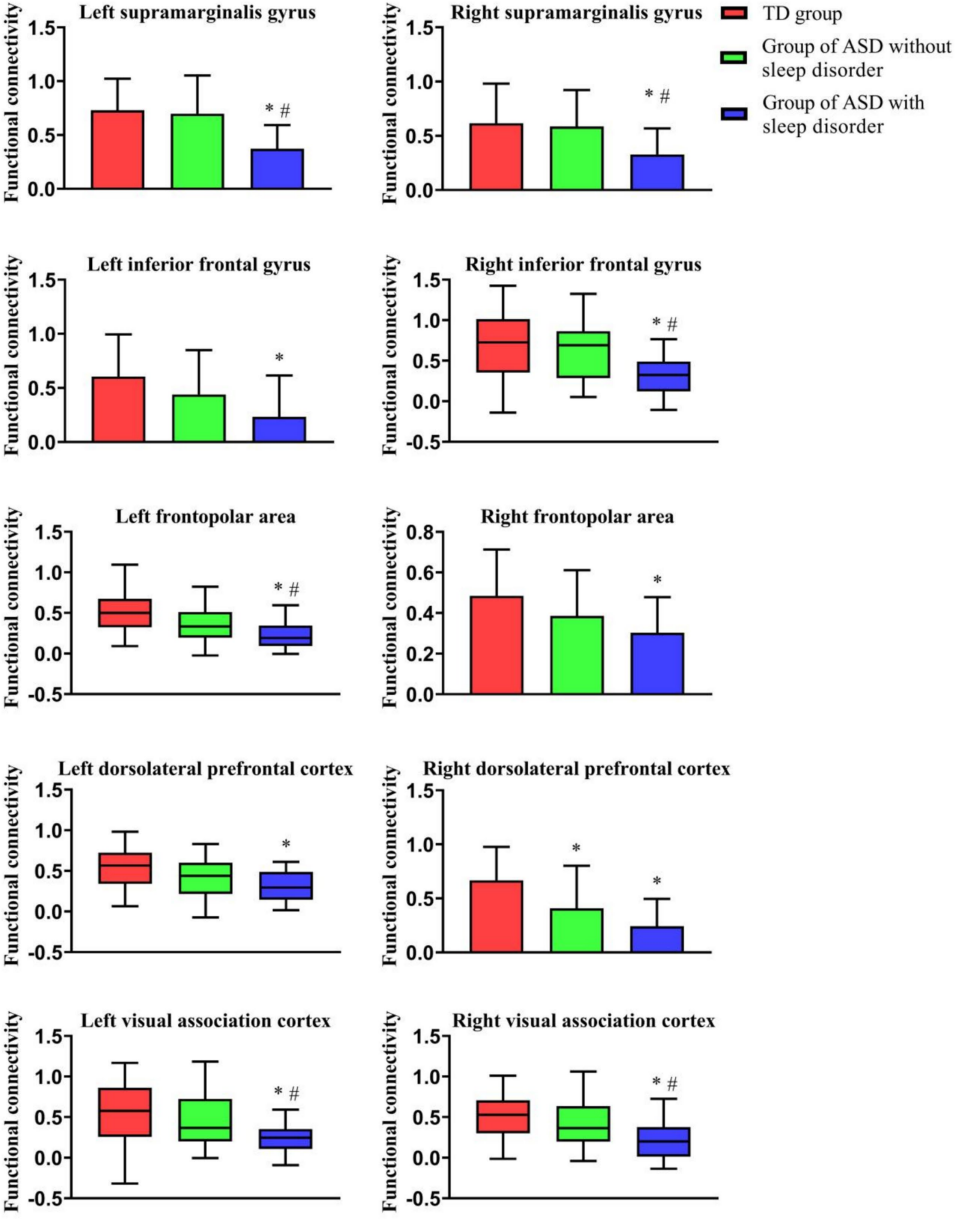
Comparison of FC among the three groups of children under resting-state conditions. This figure shows the FC of the three groups in different ROI regions; ^*^indicates a comparison with the TD group, *p* < 0.01; and ^#^indicates a comparison with the ASD without sleep disorder group, *p* < 0.01.

**Figure 3:**
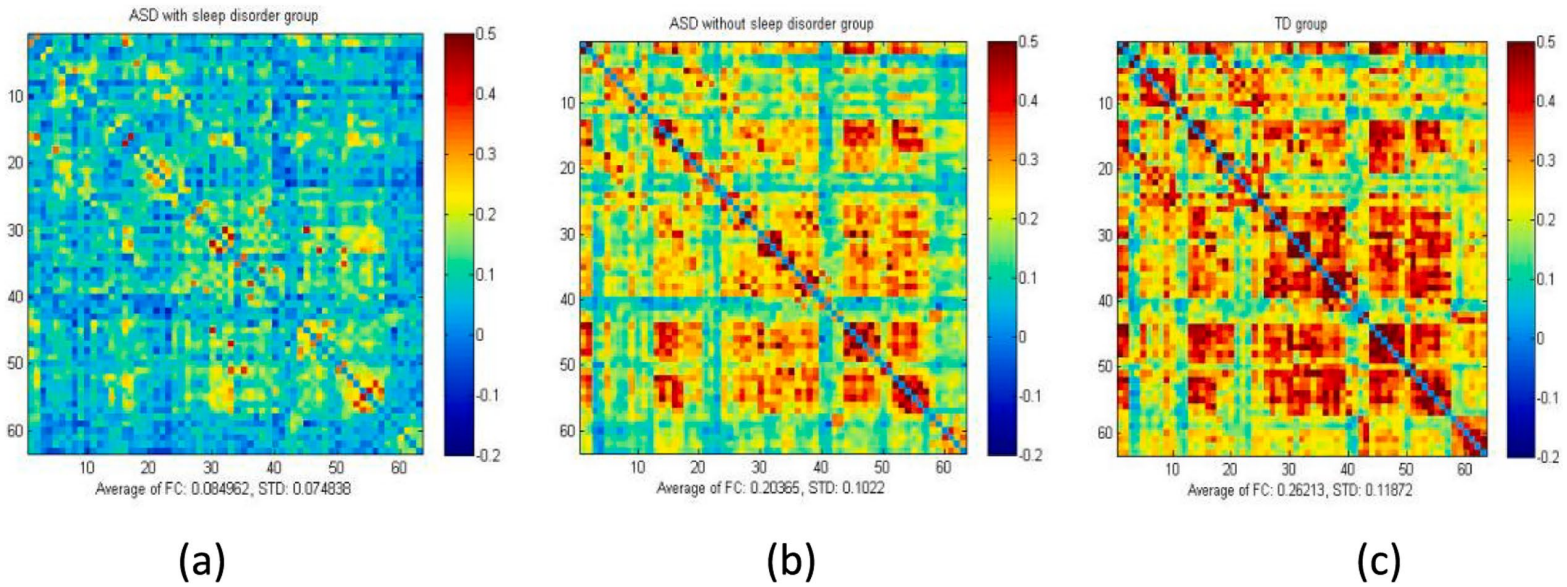
Average FC values of the three groups of children under resting-state conditions. The figure presents the overall mean matrix of the three groups under resting-state conditions: **(a)** ASD with sleep disorder group, **(b)** ASD without sleep disorder group, and **(c)** the TD group. The two axes indicate different brain regions. The correlation coefficient for each channel was set to zero (diagonal).

Table 2 shows the results of the comparisons of brain functional FC among the three groups of children under resting-state conditions. Data from three groups were analyzed using ANOVA, revealing significant differences between the groups (*p* < 0.05). Children in the ASD without sleep disorder group showed lower FC values in the IFPA than in the TD group, but no significant differences were seen in other brain regions. The SMG, IFG, FPA, DLPFC, and VAC were found to have significantly lower FC values in the ASD with sleep disorder group relative to the TD group (*p* < 0.05). The FC values for the SMG, RFPA, DLPFC, and VAC were all lower in the ASD with sleep disorder group than in the ASD without sleep disorder group. There were no significant differences between the IFG and IFPA in terms of sleep disorders. The effect sizes exceeded the large criterion (Cohen’s *f* > 0.4), indicating substantial between-group differences. Post-hoc tests confirmed that the ASD with sleep disorder group had the lowest strength of connection (*p* < 0.05) (Figures 2, 3).

**Table 2:** Comparison of FC among the three groups of children under resting-state conditions.

Brain area	TD group (30)	Group of ASD without sleep disorder (29)	Group of ASD with sleep disorder (29)	Statistics	*p*-value	Cohen’s *f*
LSMG	0.73 ± 0.29	0.57 ± 0.23	0.19 ± 0.17^*#^	40.87	<0.001	0.981
RSMG	0.61 ± 0.40	0.47 ± 0.32	0.24 ± 0.25^*#^	9.28	<0.001	0.467
LIFG	0.55 ± 0.35	0.35 ± 0.41	0.20 ± 0.30^*^	7.33	0.002	0.415
RIFG	0.60 ± 0.37	0.42 ± 0.38	0.25 ± 0.24^*^	8.33	<0.001	0.443
LFPA	0.48 ± 0.28	0.24 ± 0.20^*^	0.16 ± 0.16^*^	16.34	<0.001	0.620
RFPA	0.47 ± 0.25	0.33 ± 0.19	0.18 ± 0.11^*#^	17.10	<0.001	0.634
LDLPFC	0.57 ± 0.32	0.40 ± 0.23	0.22 ± 0.16^*#^	14.95	<0.001	0.593
RDLPFC	0.61 ± 0.43	0.44 ± 0.38	0.13 ± 0.28^*#^	12.74	<0.001	0.547
LVAC	0.57 ± 0.42	0.38 ± 0.34	0.17 ± 0.18^*#^	10.64	<0.001	0.500
RVAC	0.47 ± 0.38	0.39 ± 0.27	0.12 ± 0.20^*#^	11.65	<0.001	0.524

^*^Indicates a comparison with the TD group, *p* < 0.01; ^#^indicates a comparison with the ASD without sleep disorder group, *p* < 0.01.

### 3.3 Correlation analysis between the FC of different brain regions and the different scales used for evaluation in the ASD with sleep disorder group under resting-state conditions

Table 3 shows the results of the correlation analysis between the FC of different brain regions and the various scales used for evaluation in the ASD with sleep disorder group. The IIFG FC of Social Affect (SA) scale showed a certain degree of negative correlation (r = −0.34, *p* = 0.07). The FC in the RDLPFC exhibited a certain degree of negative correlation with restricted repetitive behavior (RRB) (r = −0.41, *p* = 0.03). The social reactivity scale scores were negatively correlated to the FC in the RVAC (r = −0.45, *p* < 0.01). The scores of the Children’s Sleep Habits Questionnaire were positively correlated with FC levels in the RIFG regions of the brain (r = 0.37, *p* = 0.05).

**Table 3:** FC value of each brain region and its correlation with the scores of each scale in the ASD with sleep disorder group.

Brain area		Social affect (SA)	Restricted repetitive behavior (RRB)	Social reactivity scale (SRS)	Children’s sleep habits questionnaire (CSHQ)
LSMG	r	0.15	−0.06	0.28	0.12
	*p*-value	0.42	0.76	0.14	0.54
	Sample size	29	29	29	29
RSMG	r	0.06	−0.19	−0.06	0.01
	*p*-value	0.75	0.32	0.77	0.96
	Sample size	29	29	29	29
LIFG	r	−0.34^*^	−0.14	−0.25	−0.15
	*p*-value	0.07	0.48	0.18	0.44
	Sample size	29	29	29	29
RIFG	r	−0.20	0.02	−0.30	0.37^*^
	*p*-value	0.29	0.91	0.11	0.05
	Sample size	29	29	29	29
LFPA	r	0.09	−0.12	0.04	−0.13
	*p*-value	0.64	0.53	0.85	0.49
	Sample size	29	29	29	29
RFPA	r	0.06	−0.28	0.19	−0.28
	*p*-value	0.76	0.14	0.32	0.14
	Sample size	29	29	29	29
LDLPFC	r	0.12	−0.28	−0.10	0.20
	*p*-value	0.53	0.14	0.61	0.29
	Sample size	29	29	29	29
RDLPFC	r	−0.08	−0.41^*^	−0.16	−0.35
	*p*-value	0.69	0.03	0.41	0.06
	Sample size	29	29	29	29
LVAC	r	−0.10	−0.26	−0.33	0.06
	*p*-value	0.59	0.18	0.08	0.75
	Sample size	29	29	29	29
RVAC	r	−0.00	−0.09	−0.45^**^	0.03
	*p*-value	1.00	0.66	0.01	0.86
	Sample size	29	29	29	29

^*^p < 0.05, ^**^p < 0.01.

## 4 Discussion

This study aimed to investigate differences in cortical FC, especially in brain regions associated with sleep disorders, in children with ASD with sleep disorders compared with ASD children without sleep disorders and TD children using fNRIS monitoring, as well as to evaluate possible correlations between ROI and sleep disorders. To prevent the influence of confounding factors, three groups of children with similar ages and sex distribution were compared.

We found that FC was lower in both ASD groups, with and without sleep disorders, than in the TD group, with the ASD sleep disorder group having significantly lower values than the TD group. Furthermore, the FC in the SMG, RFPA, DLPFC, and VAC was lower in ASD children and sleep disorders compared to those with ASD without sleep disorders, but there were no significant differences in FC in the IFG or LFPA between the two ASD groups.

Many studies to date have focused on the FC in different brain regions (Just et al., 2012). Neuroimaging and genetic studies have confirmed the heterogeneity of ASD (Jones and Klin, 2009; Klin et al., 2002; Qiu et al., 2022), with differences appearing in the early stages of development, such as differences in stereotyped behavior and eye contact. An fNIRS study found that the bilateral temporal resting-state FC was weaker in ASD children than in TD children. In children with ASD, FC levels in the right prefrontal cortex and bilateral prefrontal cortex were reported to be significantly reduced when performing demanding cognitive tasks (Chan et al., 2022). This was consistent with the findings of our study.

Previous studies have demonstrated that children with ASD have abnormal neural connections in the brain, and their abnormal pathophysiological mechanisms might be the direct cause of sleep disorders in these children (Missig et al., 2020). The RDLPFC is responsible for the action of verbal/auditory and spatial information in the brain. It is an important part of the integrated distribution of brain networks. In children with ASD, DLPFC is dysfunctional (Herrington et al., 2015; Solomon et al., 2014). It was found that prefrontal brain FC in children with ASD with sleep disorders was less than that in TD children, i.e., in brain regions associated with sleep disorders (e.g., the default mode network, prefrontal cortex), suggesting that this reduced connectivity may be linked to sleep disorders.

The prefrontal cortex, which contains the inferior and superior frontal gyrus, is involved in emotional regulation and the coordination of cognitive control and emotional regulation, thereby assisting in the control and regulation of emotional responses. Hadjikhani and Asberg Johnels (2023) reported that the mirror neuron system brain regions, which are closely related to language function in the inferior frontal gyrus, inferior parietal lobule, and superior temporal sulcus of children with ASD, have thinner cortex and lower FC than other brain regions. The associations between the FC of individual brain regions with the scores of different scales were assessed in children with autism combined with sleep disorders, showing a negative correlation between the social response score and FC in the right inferior frontal gyrus. The Repetitive Stereotyped Behavior Scale score was also negatively correlated with FC in the right dorsolateral prefrontal lobe to a certain extent. However, the scores of the Children’s Sleep Habits Questionnaire were observed to be positively correlated with FC in the right inferior frontal gyrus FC to some extent, differing from the findings of previous studies. Considering that the inferior frontal gyrus is not directly involved in the core brain area responsible for sleep–wake regulation, but only indirectly affects the development of sleep disorders through emotional regulation, cognitive control, language processing, and the default mode network, further clinical verification is needed.

The pathogenesis of sleep disorders in children with ASD is unclear and has been shown to have a substantial impact on children’s quality of life, with ASD children often having more severe language and social deficits, problematic behaviors, and emotional issues than children with ASD who do not have sleep disorders (Mazurek et al., 2019), with an association between the severity of sleep disorders and core clinical symptoms. Behavioral problems also exacerbate sleep disorders, and there may be an interaction between the two aspects (Hunter et al., 2021; Tse et al., 2019).

Shen et al. observed reduced connectivity between the primary visual cortex and sensorimotor areas in preschoolers, which was associated with sensory hypersensitivity, which is consistent with the findings of the present study.

The recruitment of children with ASD in this study was limited to rehabilitation centers, where participants may have had more pronounced clinical symptoms and comorbidities, resulting in a sample more skewed toward moderately severe patients. This may introduce bias if generalized to the broader ASD population. The TD group, however, was recruited through multiple channels, and while it encompassed a wider range of typically developing individuals, it did not match the ASD group in terms of economic, educational, and other variables, potentially introducing confounding factors. Differences in recruitment methods may impact the generalizability of the study’s findings.

## 5 Limitations

In this study, we found that the prefrontal lobes of children with ASD exhibit reduced FC. However, because FNIRS only detects changes in the cerebral cortex and is unable to assess the activities of deep subcortical structures, investigation of the brainstem and hypothalamus, which regulate sleep, remains limited. Further studies combining functional magnetic resonance imaging, magnetoencephalography, and electroencephalography to provide multimodal imaging are needed, as these would be more effective in investigating both cortical and subcortical activity. Some children with ASD were unable to sit quietly during the fNIRS examination, so they were asked to sit quietly for 3 h before the start of the examination, with the assessor providing reassurance (e.g., counting numbers and singing children’s songs) if necessary and excluding interfering factors such as medication use. This study may have underrepresented patients with mild ASD due to the single-center limitation. Future studies should focus on further optimizing the sampling design to ensure a more representative sample. In addition, the sample size of the study is relatively small; future studies should increase the sample size, use multimodal imaging, and further explore the associations between the hypothalamus, amygdala, cingulate gyrus, and other regions and sleep disorders.

## 6 Conclusion

The fNIRS technology facilitates the measurement of cerebral hemodynamic changes in the activation degree, tissue status, and FC in different brain regions in children with ASD with sleep disorders. The FC in SMG, IFG, FPA, DLPFC, and VAC were inferior in children with ASD with sleep disorder compared to TD children. However, the small sample size of this study, the cross-sectional investigation of cerebral cortical development in children with ASD and sleep disorders, the absence of EEG monitoring of sleep status, and the limitation of single-center recruitment all indicate that the generalizability of the findings needs further validation through multicenter, large-sample studies.

## Data Availability

The datasets presented in this study can be found in online repositories. The names of the repository/repositories and accession number(s) can be found in the article/supplementary material.
